# Impact of clinical stage and location of dental caries lesions on oral health–related quality of life

**DOI:** 10.1590/1807-3107bor-2025.vol39.125

**Published:** 2025-12-01

**Authors:** Shyrley DÍAZ, Jenny ABANTO, Marcelo BONECKËR, J. Sebastian LARA, Saul Martins PAIVA, Isabel C OLEGÁRIO

**Affiliations:** (a) Universidad de Cartagena, Dental School, Department of Preventive and Community Dentistry, Cartagena, Colombia.; (b) International University of Catalunya, School of Dentistry, Paediatric Dentistry Department, Barcelona, Spain.; (c) Universidade de São Paulo – USP, School of Dentistry, Department of Paediatric Dentistry and Orthodontics, São Paulo, SP, Brazil.; (d) Indiana University School of Dentistry, Department of Cariology, Operative Dentistry and Dental Public Health, Indianapolis, IN, United States.; (e) Universidade Federal de Minas Gerais – UFMG, School of Dentistry, Department of Paediatric Dentistry, Belo Horizonte, MG, Brazil.; (f) RCSI University of Medicine and Health Sciences, School of Dentistry, Dublin, Ireland.

**Keywords:** Dental caries, Oral Health, Quality of Life, Child

## Abstract

Assess the association between dental caries clinical stage, lesion location, and oral health-related quality of life (OHRQoL) in preschool children. A cross-sectional study was conducted with a representative sample of 643 caregiver–child dyads aged 0–5 years. Children underwent oral examinations using the International Caries Detection and Assessment System (ICDAS) and the International Caries Classification and Management System (ICCMS™) criteria to classify caries stage and lesion location. Traumatic dental injuries (TDI) were assessed using Andreasen’s classification. Caregivers completed the Early Childhood Oral Health Impact Scale (ECOHIS). Weighted Kappa coefficients assessed intra- and inter-examiner reliability. Negative binomial regression models, adjusted for sociodemographic variables and clinical factors, estimated the association between caries stage/location and ECOHIS scores. The prevalence of dental caries (all stages) was 72.6%, with 27.3% of children caries-free. Overall, 48.2% of caregivers reported at least one oral health impact in their child’s life (ECOHIS ≥ 1). Children with extensive lesions (stage C) had significantly worse total and domain ECOHIS scores than caries-free children (p < 0.001), regardless of lesion location. Moderate lesions (stage B) affected OHRQoL only when present in both anterior and posterior teeth (RR = 1.54; p = 0.045). Older age, public school attendance, and household crowding were also associated with poorer OHRQoL. The presence of TDI was independently associated with poorer OHRQoL (RR = 1.63; p < 0.001). These findings highlight the need for targeted, patient-centred interventions to address caries burden effectively.

## Introduction

Early childhood caries (ECC) affects nearly half of preschool children worldwide.^1^ The reported prevalence of dental caries varies depending on the diagnostic criteria applied, as caries detection involves recognising the signs and symptoms of the disease at different levels of severity.^2^ The International Caries Classification and Management System (ICCMS™)^3^ categorises caries lesions according to severity—sound, initial, moderate, and extensive—by integrating scores from the International Caries Detection and Assessment System (ICDAS). Accurate clinical detection of caries lesions is essential for epidemiological studies, as it provides reliable estimates of disease prevalence within a population. Such information is critical for informing evidence-based policies and designing targeted strategies for caries prevention and management.^4^

Severe ECC can lead to significant consequences, including pain, infection, sepsis, impaired growth and development, and increased days with restricted activity, ultimately exerting a substantial negative effect on a child’s oral health–related quality of life (OHRQoL).^5^ The impact of dental caries on OHRQoL in children has been widely investigated.^6-11^ For preschool-aged children, the Early Childhood Oral Health Impact Scale (ECOHIS)^12^ is a validated instrument that measures the effects of oral health problems on both the child and their family. ECOHIS captures the broader implications of ECC, such as difficulties in eating, speaking, sleeping, and social interaction, which may not be apparent in clinical examinations alone. The instrument comprises two main sections: the Child Impact Section—divided into four domains (symptoms, function, psychology, and self-image/social interaction)—and the Family Impact Section, which includes parental distress and family function.

Most epidemiological studies examining the relationship between OHRQoL and dental caries have focused exclusively on cavitated lesions, typically using the dmft index (decayed, missing, and filled teeth) as the main exposure measure. However, research evaluating the impact of different stages of caries severity on OHRQoL remains limited, and even fewer studies have considered the influence of lesion location (anterior versus posterior teeth).^10,11^

Incorporating both clinical and quality-of-life assessments—including lesion severity and location—can support the development of more comprehensive, patient-centred care plans that address the full spectrum of needs associated with ECC.^11^ Therefore, the aim of this cross-sectional study was to investigate the impact of dental caries, classified by clinical stage of severity and lesion location, on the OHRQoL of Colombian preschool children, as measured by the ECOHIS. We hypothesised that both greater severity of caries lesions and their presence in anterior teeth would be associated with poorer OHRQoL.

## Methods

### Study design

A cross-sectional analytical study was conducted to evaluate the association between the clinical stage and location of dental caries lesions and oral health–related quality of life (OHRQoL) in preschool children. The study adhered to the Strengthening the Reporting of Observational Studies in Epidemiology (STROBE) guidelines.

### Setting

The research was carried out in public and private preschools in Cartagena, Colombia. Data collection occurred between February and September 2022. Clinical examinations and interviews with caregivers took place on school premises during regular hours, in rooms adapted to ensure adequate lighting, ventilation, and privacy.

### Participants

The study population comprised caregiver–child dyads. Children were eligible if they were aged 3–5 years, enrolled in participating preschools, and in good general health. Exclusion criteria included children with systemic diseases or developmental disorders affecting the craniofacial region, those undergoing orthodontic or extensive restorative treatment, and those whose caregivers were not available to complete the questionnaire. Participants were recruited through an invitation letter sent to parents or legal guardians, followed by verbal explanations provided during school meetings. Written informed consent was obtained from all participating caregivers prior to enrolment.

### Variables

The primary outcome variable was OHRQoL, assessed using the validated Colombian Spanish version of the Early Childhood Oral Health Impact Scale (ECOHIS). The main exposure variables were caries lesion severity stage and lesion location, assessed according to the International Caries Classification and Management System (ICCMS™) criteria. Caries lesions were classified as: Sound (score 0), Initial (score A), Moderate (score B) and Extensive (score C) (Table 1).

**Table 1:** Classification of the staging of caries - ICCMS™ Caries Scoring System.^3^

ICCMS™ Caries Scoring System	Description
0 - Sound	Sound surfaces have no visible caries when viewed clean and dry (ICDAS™ code 0). Developmental defects like enamel hypoplasias; fluorosis; tooth wear (attrition, abrasion and erosion), and extrinsic or intrinsic stains should be recorded as sound in the absence of other signs of caries lesions.
A - Initial	Initial stage caries is characterized by the first visual change in enamel (seen only after prolonged air-drying) (ICDAS™ code 1) or a distinct visual change in enamel (seen on a wet or dry surface) (ICDAS™ code and 2). Initial active stage lesions on free smooth surfaces are located in close proximity (in touch or within 1 mm) to the gingival margin or adjacent to orthodontic or prosthetic attachments on a tooth surface.
B - Moderate	Moderate stage caries is characterized visually by either localized enamel breakdown (without visual signs of dentinal exposure) (ICDAS™ code 3) or an underlying dark shadow from dentin (ICDAS™ code 4). Enamel breakdown (ICDAS™ code 3) is often viewed best when the tooth is air-dried, whilst shadowing from dentinal caries (ICDAS™ code 4) is often best seen with the tooth surface wet.
C - Extensive	Extensive stage caries is characterised by distinct cavitation exposing visible dentin. (ICDAS™ code 5 lesions exhibit cavitation involving less than half the tooth surface and ICDAS™ code 6 involves half of the tooth surface or more).

Lesion location was categorised as affecting anterior teeth only, posterior teeth only, or both anterior and posterior teeth. Potential confounders included sociodemographic factors (child’s age, sex, school type, household crowding) and the presence of traumatic dental injuries (TDI).

### Data sources and measurement

OHRQoL was measured using the ECOHIS, which consists of 13 items: nine in the Child Impact Section (symptoms, function, psychological well-being, self-image/social interaction) and four in the Family Impact Section (parental distress, family function). Caregivers rated each item on a 5-point Likert scale ranging from “never” (0) to “very often” (4); “don’t know” responses were coded as missing and replaced by the mean score of the completed items in the respective section when ≤2 responses were missing. Higher scores indicated a greater negative impact on OHRQoL.

Dental caries detection was performed by two trained and calibrated examiners using a headlamp, disposable dental mirrors, and ball-ended WHO probes, following the ICCMS™ criteria. Weighted Kappa coefficients were calculated for inter- and intra-examiner agreement prior to data collection, with values above 0.80 considered acceptable. Teeth were cleaned with gauze before examination to remove plaque and debris.

TDI was assessed according to the Andreasen classification, recording the presence or absence of injury. Sociodemographic data were obtained via caregiver questionnaire. Household crowding was defined as the ratio of total household members to the number of bedrooms, dichotomised as ≤2 or >2 persons per bedroom. School type was categorised as public or private.

### Bias

Several measures were implemented to minimise bias. Standardised examiner training and calibration reduced measurement bias in clinical assessments. The use of a validated OHRQoL instrument ensured content validity. Confounding was addressed by adjusting for relevant sociodemographic and clinical variables in the multivariable analysis.

### Study size

Sample size was calculated to detect a minimum relative risk of 1.5 for the association between caries severity and high OHRQoL impact, with 80% power and 95% confidence, assuming a 30% prevalence of high impact based on previous Colombian studies. This resulted in a minimum requirement of 600 participants.

### Quantitative variables

ECOHIS total and domain scores were analysed as continuous variables. For regression modelling, total scores were dichotomised at ≥1 versus 0 to indicate the presence of oral impacts. Caries severity was analysed both as a categorical variable (sound, initial, moderate, extensive) and in combined severity–location groups.

### Statistical methods

Descriptive statistics were computed for all variables. Bivariate analyses were conducted using chi-square tests for categorical variables and t-tests or ANOVA for continuous variables. Multivariable Poisson regression with robust variance estimation was used to estimate adjusted relative risks (RR) and 95% confidence intervals (CI) for the association between caries severity/location and OHRQoL, controlling for potential confounders (child’s age, sex, school type, household crowding, presence of TDI). Model selection was based on epidemiological relevance and statistical significance in bivariate analyses (p < 0.20). Interactions between caries severity and lesion location were tested. Missing data for ECOHIS were handled as described above, and other missing data were excluded from multivariable models using listwise deletion. Statistical significance was set at p < 0.05. Analyses were performed using Stata® version 17.0 (StataCorp, College Station, USA).

## Results

A total of 664 caregiver–child dyads were invited to participate in the study. Nine children were excluded for not meeting the eligibility criteria, resulting in 655 eligible participants. Of these, 643 returned signed informed consent forms, corresponding to a response rate of 98.2%. Most questionnaires were completed by the child’s mother (n = 540; 84.1%).

The reliability of the clinical assessments was high. Weighted kappa coefficients for inter-examiner agreement were 0.86 for ICDAS and 0.90 for TDI, while intra-examiner reliability was 0.87 for ICDAS and 0.86 for TDI.

The overall prevalence of dental caries, considering all ICCMS™ lesion stages, was 72.6% (n = 467). Only 176 children (27.3%) were caries-free. The distribution of caries by lesion stage was as follows: initial lesions (stage A), 24.9% (n = 160); moderate lesions (stage B), 13.8% (n = 89); and extensive lesions (stage C), 34.0% (n = 218). Lesions were more frequently located in posterior teeth (65.2%), followed by anterior teeth (17.5%) and both anterior and posterior teeth (17.3%) (Table 2).

**Table 2:** Prevalence of dental caries (child-level) according to stage and tooth location.

Variables	Location			Total
	Anterior teeth only (incisors/canines)	Posterior teeth only (molars)	Both anterior and posterior teeth (incisors/canines and molars)	
	n (%)	n (%)	n (%)	n (%)
Caries lesion stages				
A: Initial	36 (13.1)	129 (46.7)	111 (40.2)	276 (59.1)
B: Moderate	9 (9.7)	75 (80.7)	9 (9.6)	93 (19.9)
C: Extensive	9 (9.2)	65 (66.3)	24 (24.5)	98 (21.0)
Total	54 (11.6)	269 (57.6)	144 (30.8)	467 (100.0)

Regarding OHRQoL, 48.2% (n = 310) of caregivers reported at least one negative impact on the ECOHIS (total score ≥ 1). The prevalence of impacts in the Child Impact Section was 26.0% (n = 167), with the highest frequency reported in the symptoms domain (21.5%), followed by function (13.2%), psychological well-being (8.7%), and self-image/social interaction (5.3%). In the Family Impact Section, 9.5% (n = 61) reported impacts, predominantly in the parental distress domain (7.8%) (Table 3).

**Table 3:** Descriptive analysis of main child-related variables and ECOHIS individual and total scores.

Variables	n (%)	Oral symptoms	Functional limitations	Psychological	Self-image and social interaction	Parental distress	Family function	Total ECOHIS
		Mean (SD)	Mean (SD)	Mean (SD)	Mean (SD)	Mean (SD)	Mean (SD)	Mean (SD)
Child’s gender								
Male	310 (48.21)	0.31 (0.71)	0.67 (1.36)	0.17 (0.73)	0.10 (0.61)	0.65 (1.34)	0.22 (0.89)	2,16 (4.05)
Female	333 (51.79)	0.42 (0.80)	0,63 (1,34)	0.25 (0.73)	0,13 (0.56)	0.61 (1.30)	0.17 (0.55)	2,24 (3.85)
Child’s age (years)								
1–2	157 (24.42)	0.30 (0.75)	0.39 (1.05)	0.22 (0.72)	0.07 (0.43)	0.58 (1.33)	0.07 (0.41)	3.71 (1.75)
3–5	486 (75.58)	0.39 (0.76)	0.73 (1.42)	0.21 (0.73)	0.13 (0.62)	0.65 (1.32)	0.23 (0.81)	4.21 (2.50)
Family income (dollars month)								
≤ 518	419 (65.16)	0.39 (0.81)	0.72 (1.40)	0.24 (0.80)	0.14 (0.64)	0.58 (1.20)	0.21 (0.73)	2.30 (4.10)
> 518	224 (34.84)	0.32 (0.66)	0.53 (1.25)	0.16 (0.56)	0.08 (0.45)	0.72 (1.53)	0.17 (0.75)	2.00 (3.64)
Household crowding (members per room)								
≤ 2	476 (74.03)	0.33 (0.67)	0.49 (1.04)	0.16 (0.54)	0.09 (0.47)	0.56 (1.26)	0.20 (0.75)	1.84 (3.20)
> 2	167 (25.97)	0.44 (0.88)	0.92 (1.72)	0.31 (0.96)	0.17 (0.73)	0.76 (1.41)	0.19 (0.71)	2.80 (4.90)
Family structure								
Nuclear family	317 (49.30)	0.34 (0.73)	0.6 (1.33)	0.2 (0.72)	0.10 (0.54)	0.61 (1.28)	0.18 (0.72)	2.04 (0.75)
Non-Nuclear family	326 (50.70)	0.46 (0.85)	0.85 (1.41)	0.28 (0.76)	0.19 (0.70)	0.72 (1.45)	0.25 (0.79)	2.77 (4.19)
ICCMS staging of caries – worst criteria in the mouth								
0: No visible caries	176 (27.37)	0.30 (0.69)	0.54 (1.19)	0.19 (0.63)	0.05 (0.30)	0.53 (1.22)	0.12 (0.49)	3.48 (1.31)
A: Initial – only posterior teeth	129 (20.06)	0.28 (0.57)	0.64 (1.20)	0.10 (0.39)	0.07 (0.41)	0.48 (1.10)	1.14 (0.56)	3.66 (1.23)
A: Initial – only anterior teeth	36 (5.60)	0.33 (0.82)	0.5 (1.36)	0.27 (0.81)	0.16(0.73)	0.5 (1.02)	0.11 (0.46)	3.72 (1.63)
A: Initial – anterior + posterior	111 (17.26)	0.29 (0.61)	0.47 (1.00)	0.17 (0.61)	0.05 (0.32)	0.42 (0.95)	0.13 (0.56)	3.53 (1.46)
B: Moderate– only posterior teeth	75 (11.66)	0.36 (0.74)	0.45 (1.13)	0.12 (0.43)	0.10 (0.38)	0.65 (1.37)	0.28 (1.07)	3.81 (1.53)
B: Moderate – only anterior teeth	9 (1.40)	0.22 (0.44)	0.33 (0.70)	0 (0.00)	0.22 (0.66)	0.44 (0.88)	0.66 (1.41)	4 (2.34)
B: Moderate – anterior + posterior	9 (1.40)	0.66 (0.86)	1.33 (1.73)	0 (0.00)	0(0.00)	0.55 (1.13)	0 (0.00)	3.44 (1.01)
C: Extensive – only posterior teeth	65 (10.11)	0.69 (1.19)	1.35 (2.23)	0.64 (1.44)	0.41 (1.22)	1.44 (2.05)	0.47 (1.17)	5.95 (3.33)
C: Extensive – only anterior teeth	9 (1.40)	0.66 (0.86)	0.77 (1.39)	0.44 (0.72)	0.44 (1.33)	1.11 (1.83)	0.22 (0.66)	6.88 (5.79)
C: Extensive - anterior + posterior	24 (3.73)	0.75 (1.03)	1.12 (1.56)	0.33 (1.00)	0.29 (0.85)	1 (1.61)	0.33 (0.96)	9.04 (4.71)
ICCMS staging of caries – worst criteria in the mouth								
0: No visible caries	176(27.37)	0.30 (0.68)	0.54 (1.18)	0.19 (0.62)	0.05 (0.30)	0.53 (1.22)	0.12 (0.49)	1.75 (3.06)
A: Initial	276(42.92)	0.29 (0.62)	0.55 (1.15)	0.15 (0.55)	0.07 (0.44)	0.46 (1.03)	0.13 (0.54)	1.68 (2.92)
B: Moderate	93(14.46)	0.37 (0.73)	0.52 (1.17)	0.09 (0.39)	0.11 (0.40)	0.61 (1.29)	0.28 (1.05)	2.0 (3.35)
C: Extensive	98(15.24)	0.72 (1.13)	1.27 (2.03)	0.56 (1.31)	0.4 (1.16)	1.33 (1.94)	0.43 (1.09)	4.73 (6.70)
Traumatic dental injuries								
Absence	553 (86.00)	0.34 (0.73)	0.59 (1.27)	0.22 (0.72)	0.10 (0.49)	0.58 (1.28)	0.19 (0.76)	2.05 (3.81)
Presence	90 (14.00)	0.53 (0.92)	1 (1.74)	0.2 (0.76)	0.22 (0.96)	0.92 (1.53)	0.2 (0.58)	3.07 (4.61)

Bivariate analysis showed that children with extensive caries lesions (stage C) had significantly higher ECOHIS total and domain-specific scores compared with caries-free children (p < 0.001). Moderate lesions (stage B) were associated with higher scores only in the family function domain (p = 0.032), whereas initial lesions (stage A) were not associated with any significant differences in ECOHIS scores relative to caries-free children (p > 0.05) (Table 4).

**Table 4:** Univariate analysis of dental caries Staging associated with total and individual domains score ECOHIS.

Variables	Oral symptoms		Functional limitations		Psychological		Self-image and social interaction		Parental distress		Family function		Total ECOHIS	
	IRR (95%CI)	p-value	IRR (95%CI)	p-value	IRR (95%CI)	p-value	IRR (95%CI)	p-value	IRR (95%CI)	p-value	IRR (95%CI)	p-value	IRR (95%CI)	p-value
Caries lesion stages														
0: No visible caries (ref)														
A: Initial	0.98 (0.69–1.38)	0.905	1.02 (0.79–1.32)	0.855	0.77 (0.49–1.21)	0.263	1.50 (0.69–3.29)	0.305	0.86 (0.66–1.12)	0.264	1.11 (0.65–1.88)	0.687	0.96 (0.83–1.10)	0.558
B: Moderate	1.22 (0.80–1.88)	0.345	0.95 (0.67–1.35)	0.800	0.48 (0.23–1.01)	0.054	2.10 (0.85–5.17)	0.105	1.14 (0.82–1.58)	0.418	2.32 (1.32–4.08)	0.003*	1.13 (0.94–1.36)	
C: Extensive	2.39 (1.68–3.42)	< 0.001*	2.33 (1.79–3.05)	< 0.001*	2.89 (1.89–4.42)	< 0.001*	7,91 (3.82–16.36)	< 0.001*	2.47 (1.90–3.23)	< 0.001*	3.49 (2.08–5.86)	< 0.001*	2.69 (2.33–3.11)	< 0.001*

In the adjusted Poisson regression model (Table 5), the presence of TDI was independently associated with poorer OHRQoL (RR = 1.63; p < 0.001) and was therefore included as a covariate in all models. Extensive caries lesions, regardless of location, were significantly associated with poorer OHRQoL (RR > 2; p < 0,05) compared with caries-free children. Moderate lesions were associated with worse OHRQoL only when present in both anterior and posterior teeth (RR = 1.54; 95%CI: 1.01–2.34; p = 0.045).

**Table 5:** Poisson Unadjusted and Adjusted Regression analysis (α = 5%) of sociodemographic variables and clinical conditions associated with total ECOHIS score (unit of analysis=child n = 643).

Variables	Total ECOHIS Score			
	Unadjusted analysis		Adjusted analysis	
	RR (95%CI)	p-value	RR (95%CI)	p-value
Child age (years)				
0–2 (ref)				
3–5	1.13 (1.03–1.24)	0.008*	1.12 (1.06–1.18)	<0.001*
Child gender				
Male (ref)				
Female	1.03 (0.93–1.15)	0.500	–	–
Type of school				
Private (ref)				
Public	1.34 (1.21–1.49)	< 0.001*	1.19 (1.05–1.35)	0.004*
Family income (dollars)				
≤ 518 (ref)				
> 518	0.86 (0.77–0.97)	0.014*	–	–
Household crowding (members)				
≤ 2 (ref)				
> 2	1.35 (0.99–1.84)	0.051	1.26 (1.11–1.44)	< 0.001*
Family structure				
Nuclear family (ref)				
Non-nuclear family	1.35 (1.21–1.51)	< 0.001*	–	–
ICCMS Worst score criteria - location				
0: No visible caries (ref)				
A: Initial – only posterior	0.98 (0.82–1.16)	0.842	0.95 (0.80–1.14)	0.633
A: Initial – only anterior	1.06 (0.82–1.38)	0.618	1.09 (0.84–1.43)	0.487
A: Initial – anterior + posterior	0.88 (0.73–1.06)	0.186	0.84 (0.69–1.02)	0.069
B: Moderate– only posterior	1.11 (0.91–1.35)	0.269	0.97 (0.79–1.19)	0.802
B: Moderate – only anterior	1.06 (0.65–1.74)	0.789	0.91 (0.55–1.48)	0.701
B: Moderate: anterior +posterior	1.44 (0.94–2.20)	0.088	1.54 (1.01–2.36)	0.045*
C: Extensive – only posterior	2.84 (2.43–3.32)	< 0.001*	2.58 (2.19–3.04)	< 0.001*
C: Extensive – only anterior	2.07 (1.44–2.97)	<0.001*	1.67 (1.16–2.40)	0.005*
C: Extensive – anterior + posterior	2.16 (1.71–2.73)	<0.001*	1.85 (1.46–2.35)	<0.001*
Traumatic dental injuries (TDI)				
Absence (ref)				
Presence	1.49 (1.31–1.70)	<0.001*	1.63 (1.42–1.87)	<0.001*

Sociodemographic variables also influenced OHRQoL. Older children (3–5 years) had higher ECOHIS scores than younger children (0–2 years) (RR = 1.12; 95%CI: 1.06–1.18). Attending a public school (RR = 1.19; 95%CI: 1.05–1.35) and living in a household with >2 members per bedroom (RR = 1,26; 95%CI:1.11–1.44) were both significantly associated with greater negative impacts on OHRQoL.

## DISCUSSION

This study evaluated the impact of caries lesion stage and location on the OHRQoL of preschool children, while adjusting for relevant confounding factors, including socioeconomic characteristics^16,17^ and the presence of TDI.^9,18,19^ The findings show that extensive lesions, regardless of tooth location, were consistently associated with higher ECOHIS total and domain-specific scores, reflecting a substantial negative impact on children’s daily lives and family well-being. Moderate lesions were associated with poorer OHRQoL only when affecting both anterior and posterior teeth, whereas initial lesions had no measurable effect. Socioeconomic factors, including school type and household crowding, were also significantly associated with poorer OHRQoL.

The prevalence of ECC in this population (72.6%) was higher than the global pooled estimate of approximately 48%.^1^ However, much of this difference is attributable to the diagnostic criteria applied. The dmft index, commonly used in global prevalence studies, records only cavitated lesions, whereas the ICCMS™ criteria^3,13^ used in the present study identify both cavitated and non-cavitated lesions across the full disease spectrum. This more sensitive approach captures early-stage disease that would otherwise go unrecorded, thereby producing higher prevalence estimates while providing a more accurate picture of the true burden of caries in the population.^23,24^Traditionally, the impact of dental caries on OHRQoL has been assessed using only the most severe stages of the caries process (dmft/DMFT criteria).^9-11^ Although it is plausible to expect that the initial stages of caries lesions do not have an immediate impact on OHRQoL,^10^ non-cavitated caries lesions are more likely to progress in comparison to sound surfaces.^21,22^ Our analyses considered all stages in the caries process, as only a few studies have assessed the impact of initial and moderate caries lesions on OHRQoL in children.^10,11,21^

Lesion location emerged as an important modifier of the relationship between caries and OHRQoL. Extensive lesions in any location were detrimental to quality of life, while moderate lesions had an impact only when present in both anterior and posterior teeth. Anterior lesions may be more readily perceived by caregivers due to their aesthetic implications, potentially causing psychosocial concern for both the child and family.^20^ In contrast, posterior lesions can compromise chewing function and lead to discomfort or pain. Occlusal surfaces on primary molars and smooth surfaces on upper incisors are among the most prone to ECC.^23,24^ The observed association of moderate lesions with the family function domain suggests that the anticipated or actual treatment of such lesions may generate financial, logistical, and emotional burdens.^25^

When moderate or extensive lesions require restorative interventions, they are typically more costly and invasive than treatments for early lesions, which are often managed non- or micro-invasively. Cavitated dentine caries lesions can extend into the pulp, causing pulpitis and necrosis, which may lead to pain, infection, dietary changes, school absenteeism, and disrupted sleep.^26^ Consequently, extensive caries significantly worsened the impact on all ECOHIS domains and total scores.

Although the inclusion of initial lesions in the caries detection process did not show an impact on OHRQoL in preschool children, detecting these lesions remains essential. Early detection allows the implementation of preventive measures aimed at avoiding progression to more severe stages, which could have a higher impact on OHRQoL.^21,22^

Poorer socioeconomic indicators are generally associated with poorer quality of life.^26,27^ In the present study, school type and household crowding influenced OHRQoL scores. Children attending public schools had an increased impact on ECOHIS scores by 19%. School type has been previously used as a reliable indicator of socioeconomic status.^28^ In Colombia, children from higher-income families generally attend private schools, while children from low-income families attend public schools. Our findings suggest that OHRQoL in public school children was significantly affected, possibly reflecting a lower socioeconomic condition and limited access to public oral health services. This reveals markedly unequal burdens of the disease according to socioeconomic status. A similar trend was observed for household crowding, which may be related to unsanitary housing conditions, hostile environments, and poorer health-related habits, where oral health may not be prioritised.^29^

Several methodological strengths underpin these findings. First, the study used the validated Colombian version of the ECOHIS,^12^ ensuring cultural and linguistic appropriateness for the target population. Second, all clinical examinations were conducted by calibrated examiners with high inter- and intra-examiner agreement, reducing misclassification risk. Third, probability proportional to size sampling enhanced representativeness across public and private preschool settings in the city. Furthermore, the statistical models controlled for sociodemographic factors and TDI, which are known to influence OHRQoL.^9,16-19^

However, some limitations should be acknowledged. The cross-sectional design precludes causal inference, as temporality between caries experience and OHRQoL cannot be confirmed. The sample was limited to children attending preschool, potentially excluding children who are not enrolled and who may have greater disease severity, leading to underestimation of true population-level impacts. Caregiver-reported OHRQoL data may be subject to recall or social desirability bias, although such misclassification is likely non-differential and would bias results toward the null. Residual confounding from unmeasured factors such as diet, oral hygiene, or fluoride exposure cannot be excluded. Finally, the study was conducted in an urban Colombian setting; generalisability to rural populations or countries with different healthcare systems should be approached with caution.

From a public health perspective, these findings underscore the importance of integrated preventive and restorative strategies.^25^ Programmes should prioritise early detection of lesions, arrest of progression, and targeted resource allocation to children with more severe disease and those from socioeconomically disadvantaged backgrounds. Addressing both clinical severity and lesion location can help ensure that interventions are responsive to the specific functional and psychosocial needs of young children, ultimately improving their oral health and overall quality of life.^20^

## Conclusion

In this population of Colombian preschool children, both the severity and location of caries lesions were significantly associated with OHRQoL. Extensive lesions, regardless of tooth location, had the greatest negative impact, while moderate lesions affected OHRQoL only when present in both anterior and posterior teeth. Socioeconomic disadvantage further amplified these effects. These findings highlight the need for early detection and preventive management of all stages of ECC, with particular attention to children at greater social and economic risk, to reduce disease progression and improve the quality of life for young children and their families.

## Data Availability

The datasets generated during and/or analyzed during the current study are available from the corresponding author on reasonable request.
